# Supplementary material for: Gas diffusion enhanced electrode with ultrathin superhydrophobic macropore structure for acidic CO2 electroreduction
Source: Nat Commun. 2024 Jan 15;15:491. doi: 10.1038/s41467-024-44722-4 (PMC10789815; doi:10.1038/s41467-024-44722-4)
Supplement: Supplementary file 1 — Supplementary Information [file 41467_2024_44722_MOESM1_ESM.pdf]

Supplementary information

Gas Diffusion Enhanced Electrode with Ultrathin Superhydrophobic Macropore Structure for Acidic CO_2 Electroreduction

Mingxu Sun[1], Jiamin Cheng[2], Miho Yamauchi[1,2,3,4,5]*

[1]Department of Chemistry, Graduate School of Science, Kyushu University, Motooka 744, Nishi-ku, Fukuoka 819-0395, Japan.

[2]Research Center for Negative Emissions Technologies (K-NETs), Kyushu University, Motooka 744, Nishi-ku, Fukuoka 819-0395, Japan.

[3]Institute for Materials Chemistry and Engineering (IMCE), Kyushu University, Motooka 744, Nishi-ku, Fukuoka 819-0395, Japan.

[4]International Institute for Carbon-Neutral Energy Research (WPI-I^{2}CNER), Kyushu University, Motooka 744, Nishi-ku, Fukuoka 819-0395, Japan.

[5]Advanced Institute for Materials Research (WPI-AIMR), Tohoku University, Katahira 2-1-1, Aoba-ku, Sendai 980-8577, Japan.

*Corresponding author. Email: yamauchi@ms.ifoc.kyushu-u.ac.jp

The PDF file includes:

Supplementary Figures 1 to 37
Supplementary Tables 1 and 2
References 1 to 4

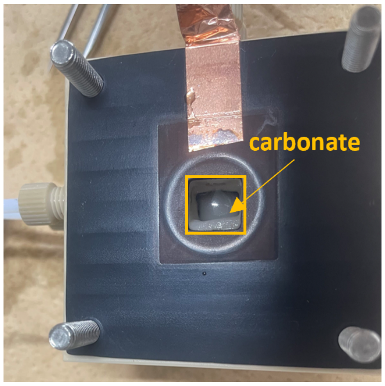

Supplementary Figure 1. Photo of the flow-type reactor after CO_2RR with long time operation in 1 M KOH electrolyte.
The yellow rectangle marks carbonate formation in the CO_2RR chamber.

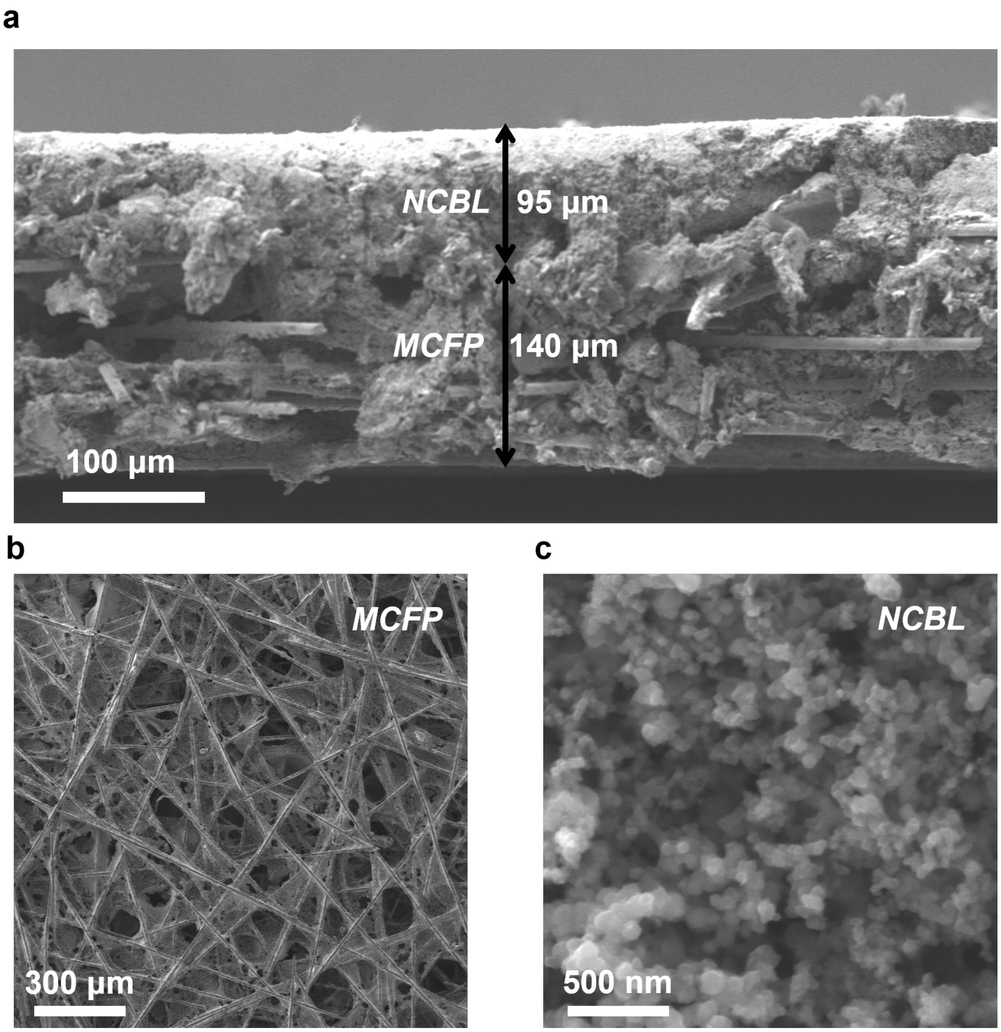

Supplementary Figure 2. SEM images of a common carbon GDL. a, cross sectional SEM image of the GDL, SEM image of **b**, MCFP and **c,** NCBL.

The most common GDL architecture is consist of macroporous carbon fiber paper (MCFP, **b**) and nano-microporous carbon black layer (NCBL, **c**). We confirmed that thicknesses of MCFP and NCBL are ~140 and ~95 μm, respectively.

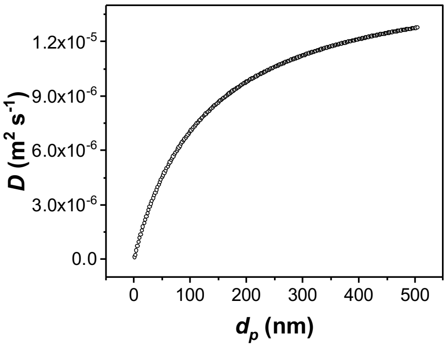

Supplementary Figure 3. Diffusion coefficient (*D*) calculated based on eq. (3) as a function of pore diameter (d_p) in the range of 0−500 nm.

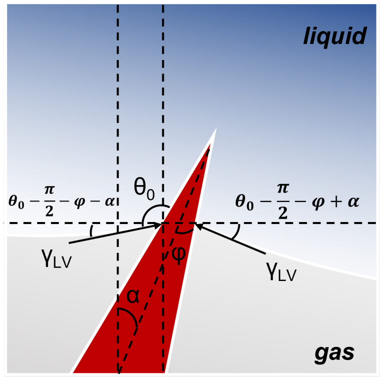

Supplementary Figure 4. Scheme of a liquid droplet on a needle-like structure.

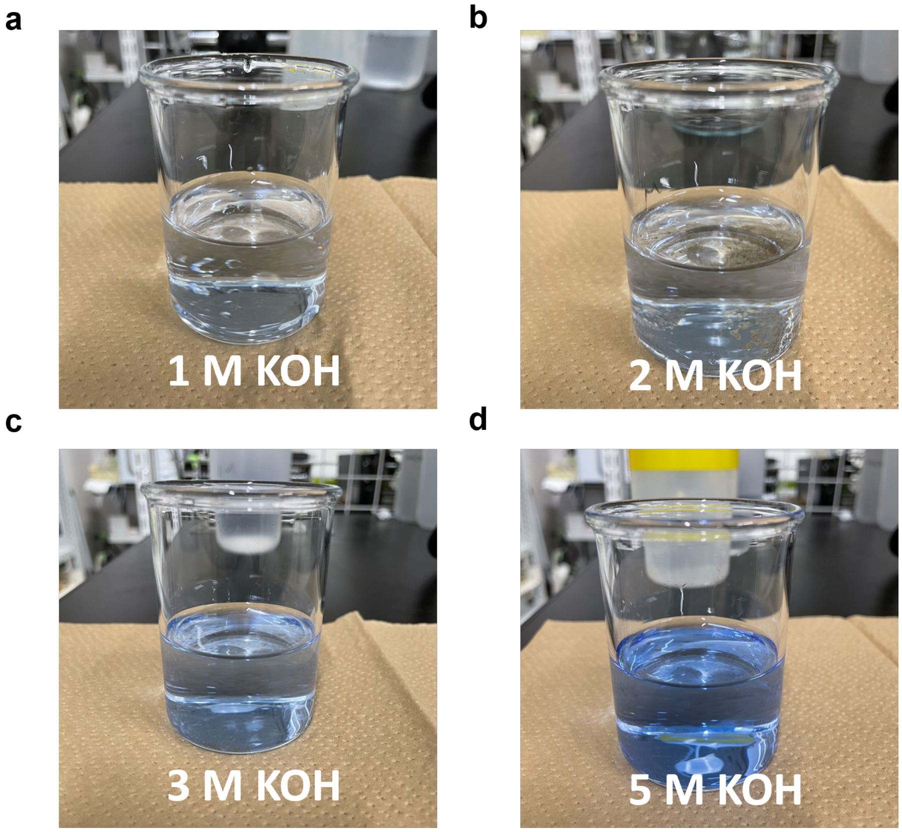

Supplementary Figure 5. Photos of different concentrations of KOH electrolyte after in situ electrooxidation of porous Cu foil at *j* of −4 mA cm^{-2}. a, 1 M, **b**, 2 M, **c**, 3 M and **d**, 5 M.

As the concentration of KOH increases, more $Cu(OH)_2$ dissolves in the electrolyte, leading to a decrease in the growth of nanometer-sized $Cu(OH)_2$ on the porous Cu foil surface.

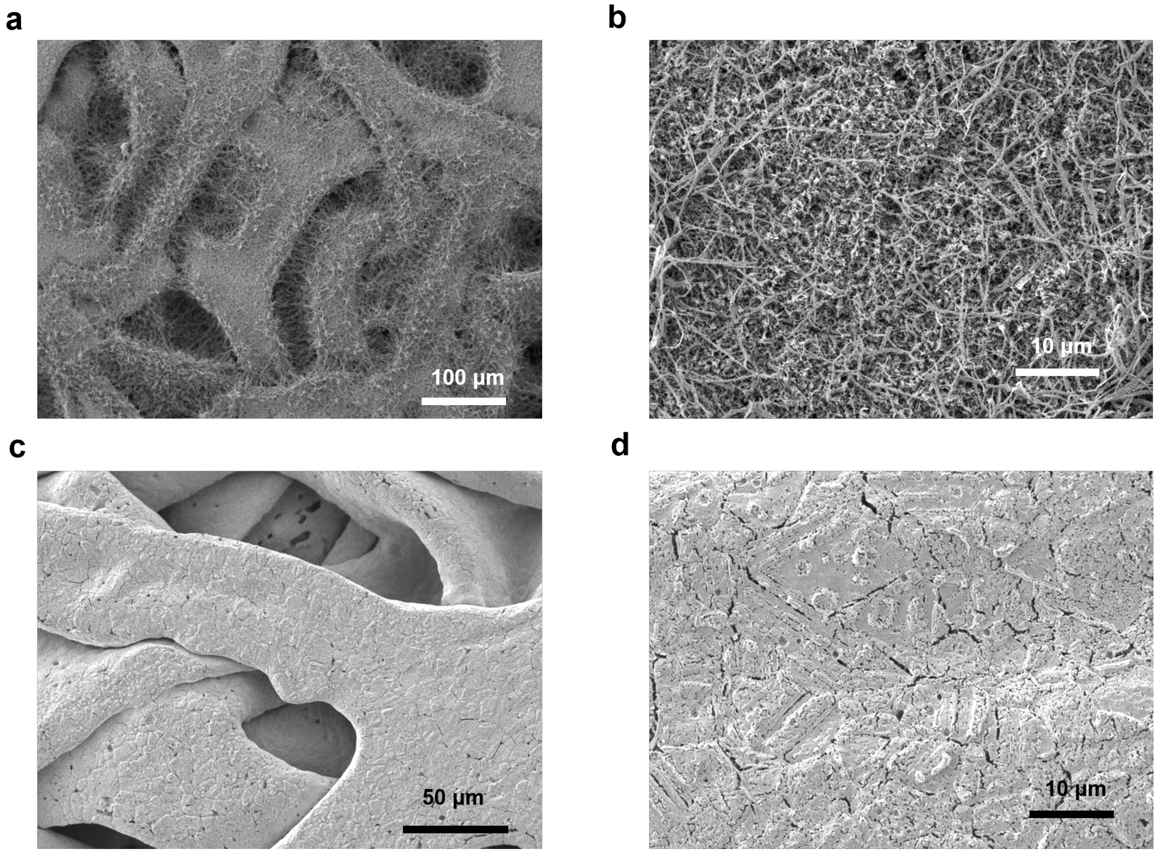

Supplementary Figure 6. SEM images of porous Cu. a,**b**, 2 M, **c**,**d**, 5 M. (In situ electrooxidation was carried out to facilitate the formation of $Cu(OH)_2$ under different concentrations of KOH. The reaction environment was maintained at 22 °C, and the electrooxidation *j* was set at −4 mA cm^{-2}).

Under 2 M KOH, $Cu(OH)_2$ can easily grow on the surface of porous Cu foil in the form of nanostructures. However, when the KOH concentration is increased to 5 M, the nanostructures grown on the surface of porous Cu foil become almost invisible. This is because the presence of synthesized $Cu(OH)_2$ in the form of dissolved electrolyte prevents the formation of nanostructures. Although a method to synthesize nanostructured Cu has been found, it does not produce the characteristic needle-like structure. In addition, some of these nanostructures may block the pores of the porous Cu foil, limiting gas diffusion.

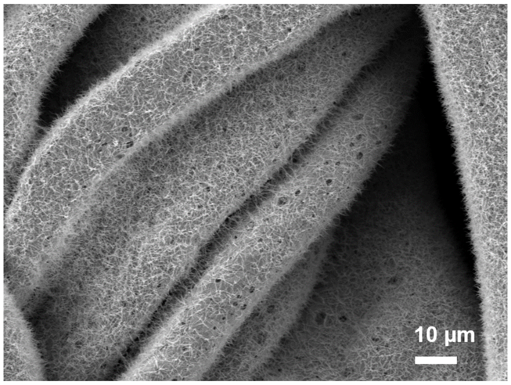

Supplementary Figure 7. SEM image of porous Cu. (In situ electrooxidation was carried out to facilitate the formation of $Cu(OH)_2$ in a reaction environment maintained at 22 °C. The electrolyte used for this process was 2 M KOH, and the electrooxidation *j* was set to −20 mA cm^{-2}).

Increasing the current density accelerates the oxidation process from Cu to Cu^{2+}, and increasing the Cu^{2+} concentration facilitates the synthesis of characteristic needle-like structures. However, the low density of needle-like nanostructures, particularly the channels, limits the contribution of superhydrophobic structures to gas diffusion.

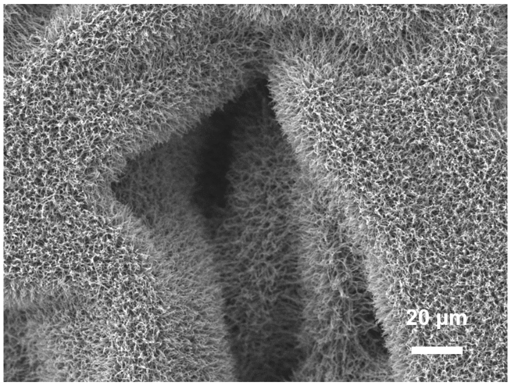

Supplementary Figure 8. SEM image of porous Cu. (In situ electrooxidation was carried out to facilitate the formation of $Cu(OH)_2$ in a reaction environment maintained at 0 °C. The electrolyte used for this process was 2 M KOH, and the electrooxidation *j* was set to −4 mA cm^{-2}).

The electrooxidation environment maintained at 0°C effectively regulates the rate of combination of Cu^{2+} and OH^{-}, enabling the growth of high-density needle-like nanostructures with no dead ends.

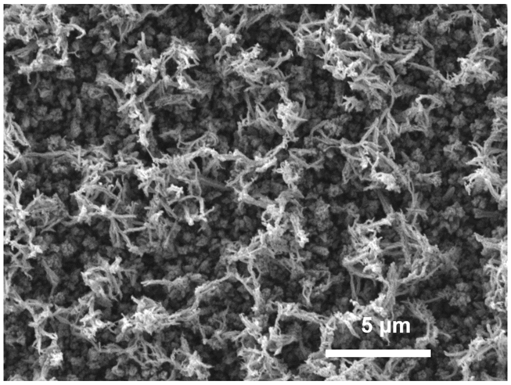

Supplementary Figure 9. SEM image of porous Cu. (The reduction of $Cu(OH)_2$ to Cu is achieved through electrochemical reduction. The electrolyte used for this process is 1M CO_2-saturated $KHCO_3$, and the reduction potential is set at −0.6 V vs RHE.)

The formation of characteristic nanostructures in porous Cu through electrochemical reduction leads to the change in the original morphology.

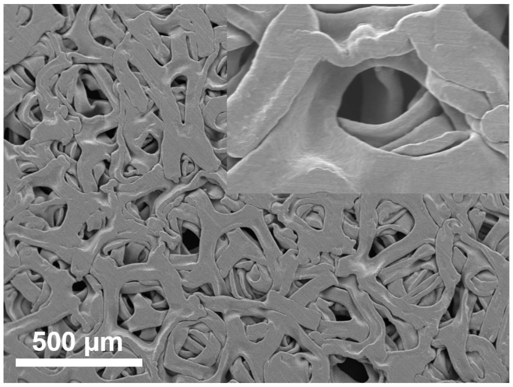

Supplementary Figure 10. SEM image of a porous Cu foil.
Porous Cu foil surface without any modification.

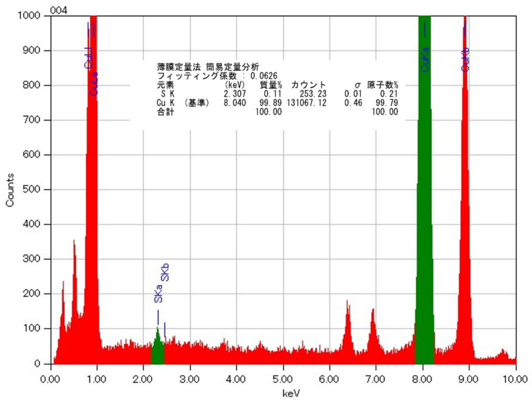

Supplementary Figure 11. Elemental composition on the surface of Cu-GDL.
The EDX measurement showed that 0.11wt.% of S is included.

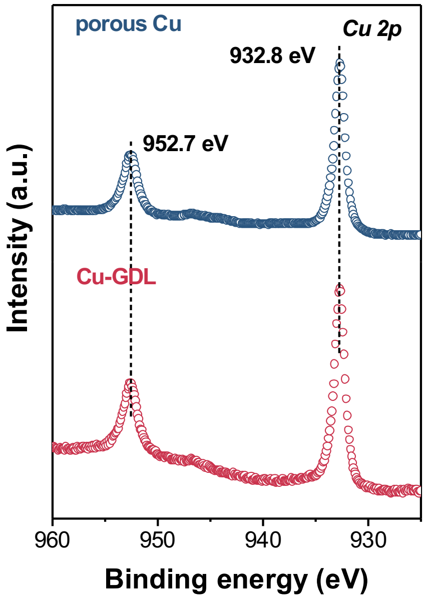

Supplementary Figure 12. XPS spectrum of Cu 2*p* for porous Cu and Cu-GDL.

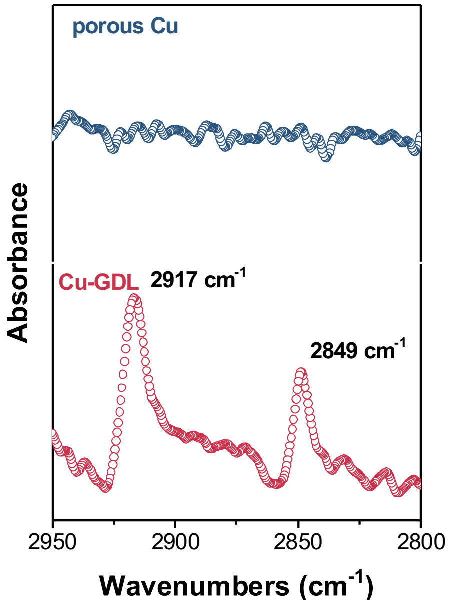

Supplementary Figure 13. ATR-FTIR spectrum for porous Cu and Cu-GDL.
The ATR-FTIR spectrum showed that the bands at 2917 and 2849 cm^{-1} are assigned to the asymmetric (ν_a) and symmetric (ν_s) CH_3 modes, respectively [1].

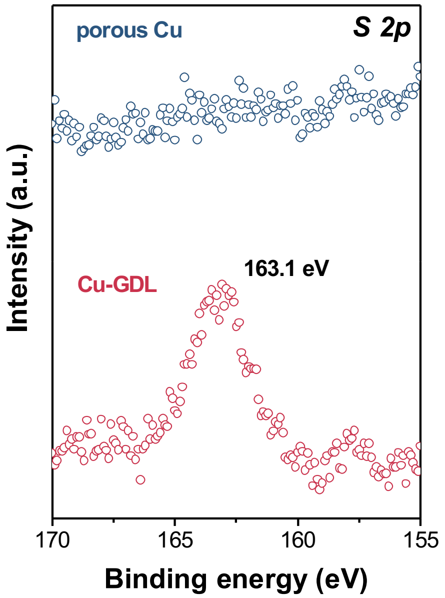

Supplementary Figure 14. XPS spectrum of S 2*p* for porous Cu and Cu-GDL.
The XPS spectrum of S 2*p* showed that the 1-octadecanethiol connects to the Cu surface through Cu–S bonds at 163.1 eV [2].

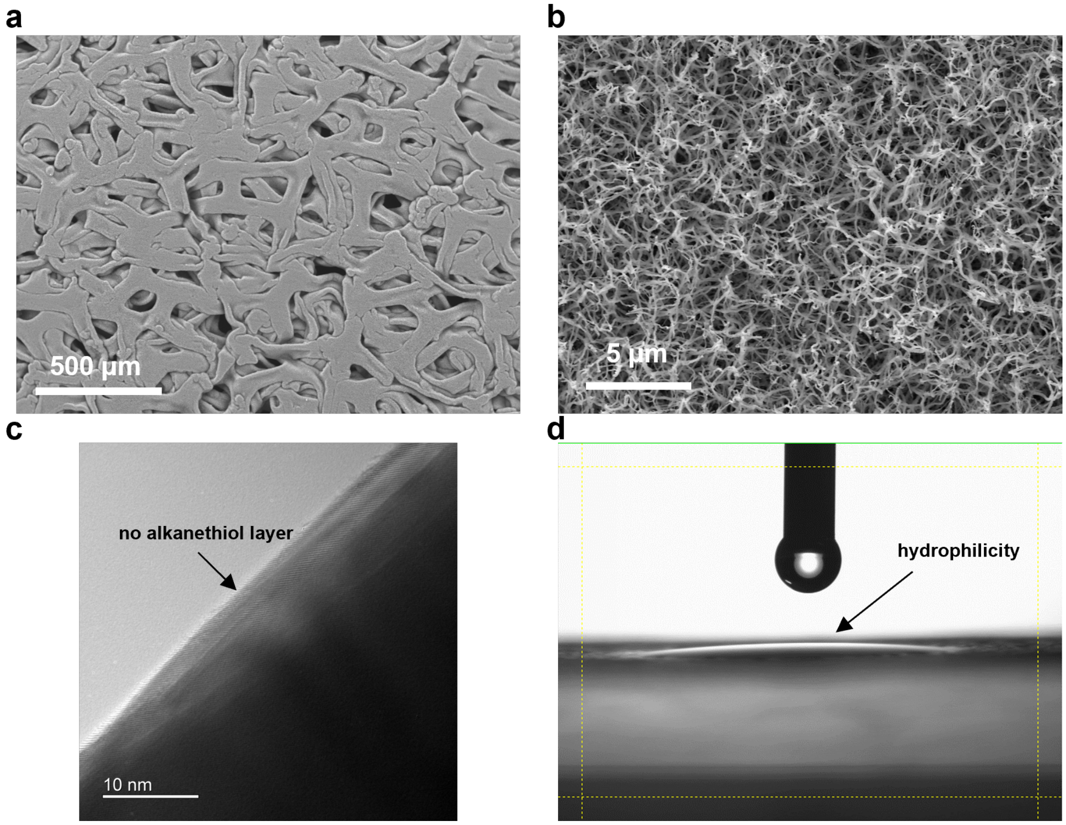

Supplementary Figure 15. Porous structure and hydrophilicity of a porous Cu. a,**b**, SEM, **c**, TEM and **d**, contact angle images.

SEM images showed that the adsorbed 1-octadecanethiol (Fig. 2c) has no influence on the electrode morphology compared to the (**b**) naked Cu surface. TEM and contact angle images showed that the naked Cu surface is hydrophilic.

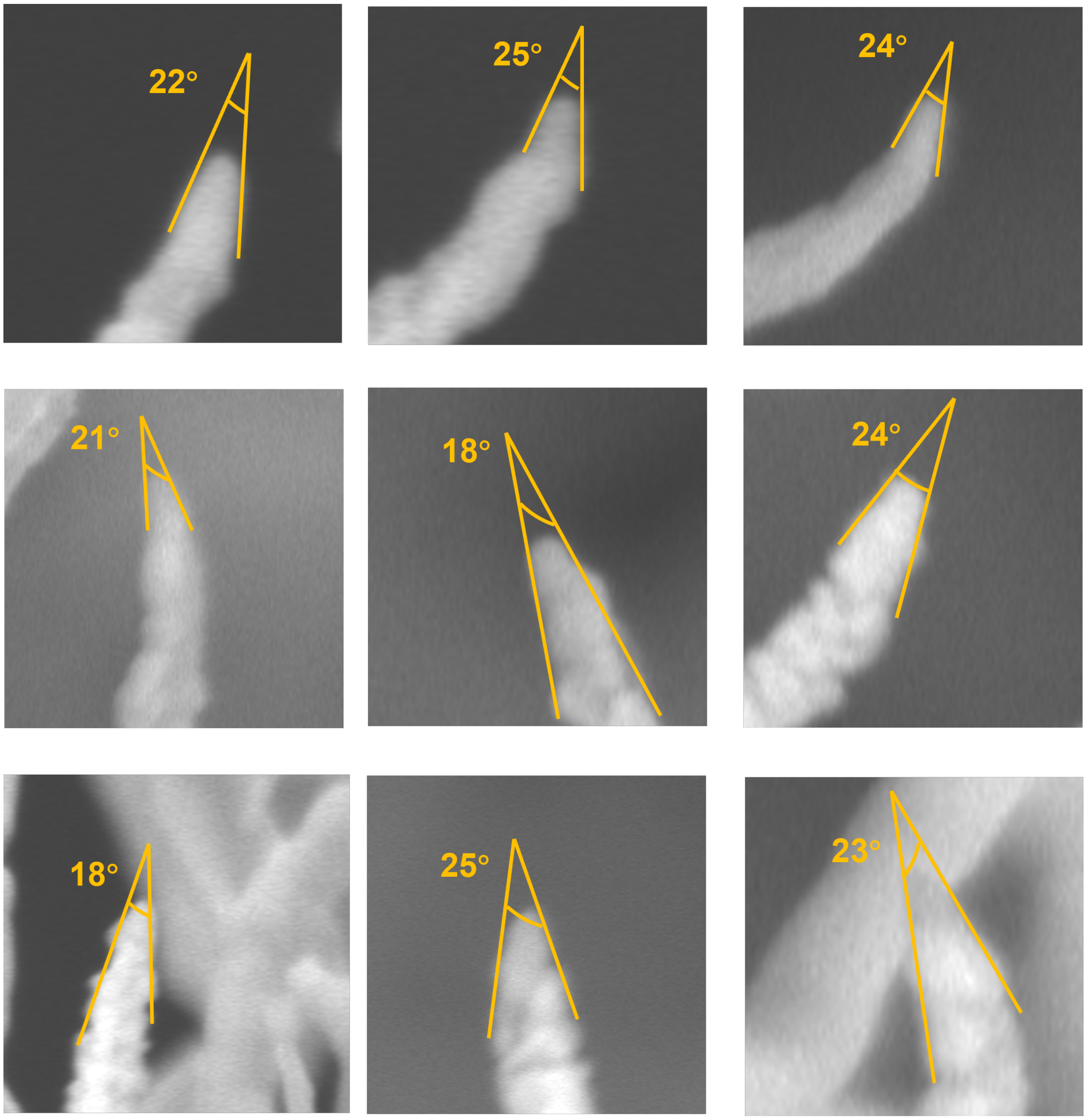

Supplementary Figure 16. Apex angles measured for Cu-GDL.
Photo images showed that the apex angles are in the range of 18–25°.

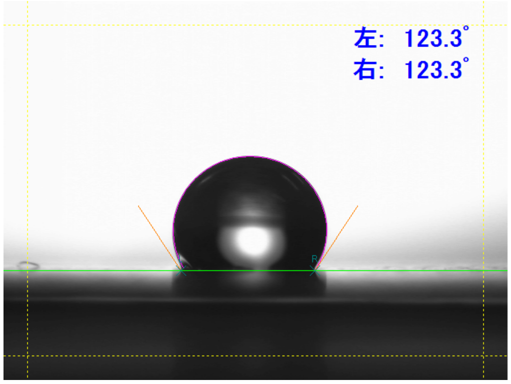

Supplementary Figure 17. Contact angle measured for a porous Cu foil coated with 1-octadecanethiol.

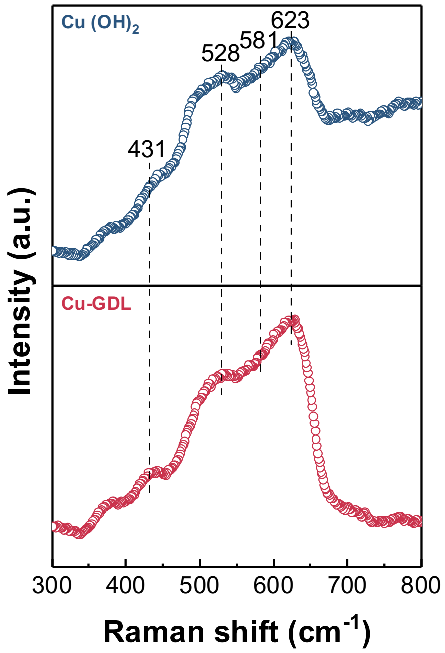

Supplementary Figure 18. Ex situ Raman spectrum for $Cu(OH)_2$ and Cu-GDL.
The Raman spectrum of $Cu(OH)_2$ and **Cu-GDL** showed the obvious peak at Raman shift of ~431, 528, 581 and 623 cm^{-1} [4].

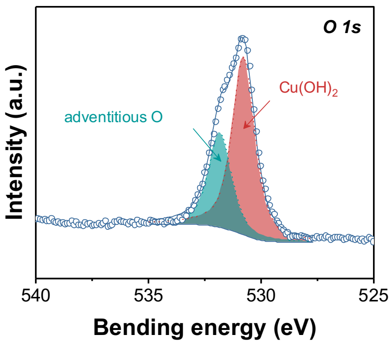

Supplementary Figure 19. XPS spectrum of O 1*s* for porous Cu.
The XPS spectrum of O 1*s* showed that a Cu surface contains O of the $Cu(OH)_2$ [3].

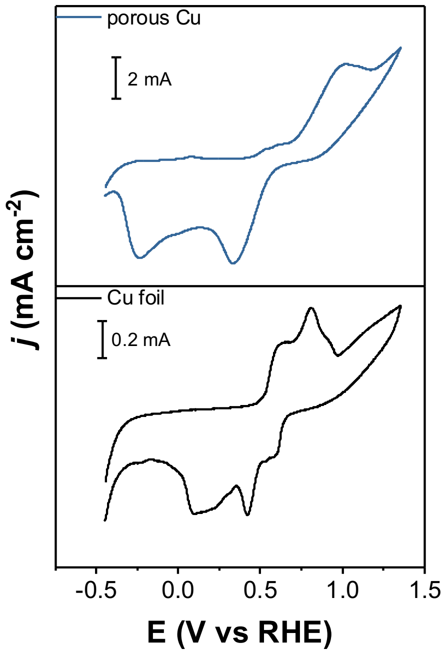

Supplementary Figure 20. Cyclic voltammograms measured for a Cu foil and porous Cu at a scan rate of 10 mV·s^{-1} in Ar-saturated 0.1 M $KHCO_3$.

The potential for oxidation of Cu^0 to Cu^1 follows a Nernst equation when the change of binding energy is negligible. Therefore, the oxidation potentials observed on any Cu metal electrodes in a same pH condition should be identical when referring RHE. However, if the pH of the electrode surface ($pH_{surface}$) is different from the pH of the bulk electrolyte (pH_{bulk}), the onset oxidation potential can change, because the potential *E* given in eq. S5 reflects the $pH_{surface}$. Thus, $pH_{surface}$ on the original Cu foil is the same as pH_{bulk} whereas *E* on porous Cu is determined by the $pH_{surface}$. It is known that the pH_{bulk} is 9.2 (Ar-saturated 0.1 M$KHCO_3$), the onset oxidation potential of a Cu foil is 0.45 V (vs. RHE), and porous Cu is 0.35 V (vs. RHE) as shown in Fig. 3 c. According to eqs. S5 and S6 we can calculate that the pH difference (ΔpH) between a Cu foil and porous Cu is 1.7, and the surface pH of porous Cu is 10.9.

$$E\text{ (vs. RHE)} = E\text{ (vs. Ag/AgCl)} + 0.209\text{ V} + 0.0592 \times pH_{surface}$$

$$pH_{surface} = pH_{bulk} + \Delta pH$$

where, $pH_{surface}$ is surface pH of the catalyst/electrode, pH_{bulk} is pH of an electrolyte.

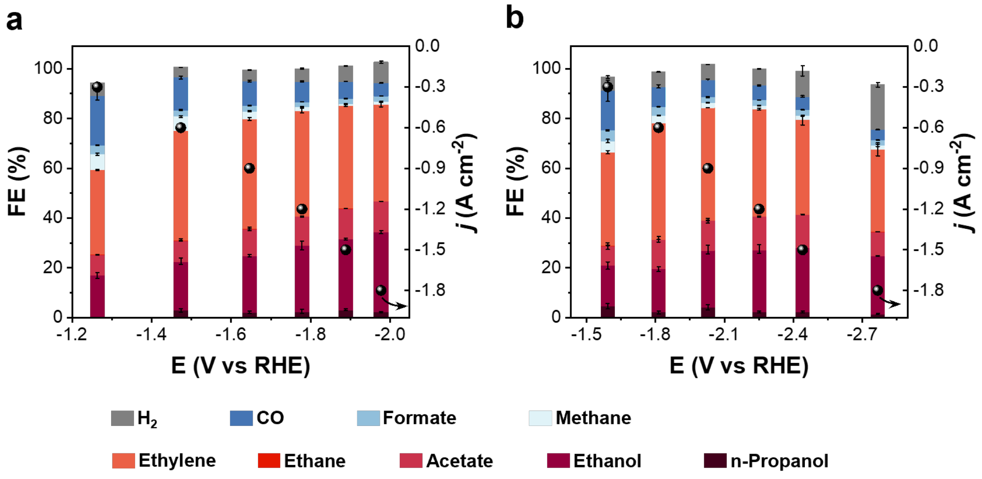

Supplementary Figure 21. CO_2RR performance of Cu-GDL with different pH electrolyte. a, pH 6, **b**, pH 1. Error bars indicate s.d. (n = 3 replicates).

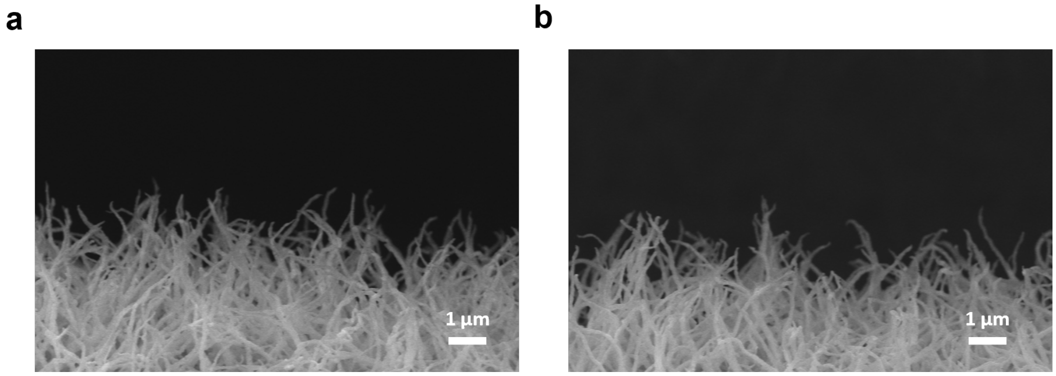

Supplementary Figure 22. Morphological characterization of Cu-GDL after operating CO_2RR at *j* from −0.3 to −1.8 A cm^{-2}. A pH 6, **B** pH 1.

Cu-GDL after operating CO_2RR maintains the original morphology.

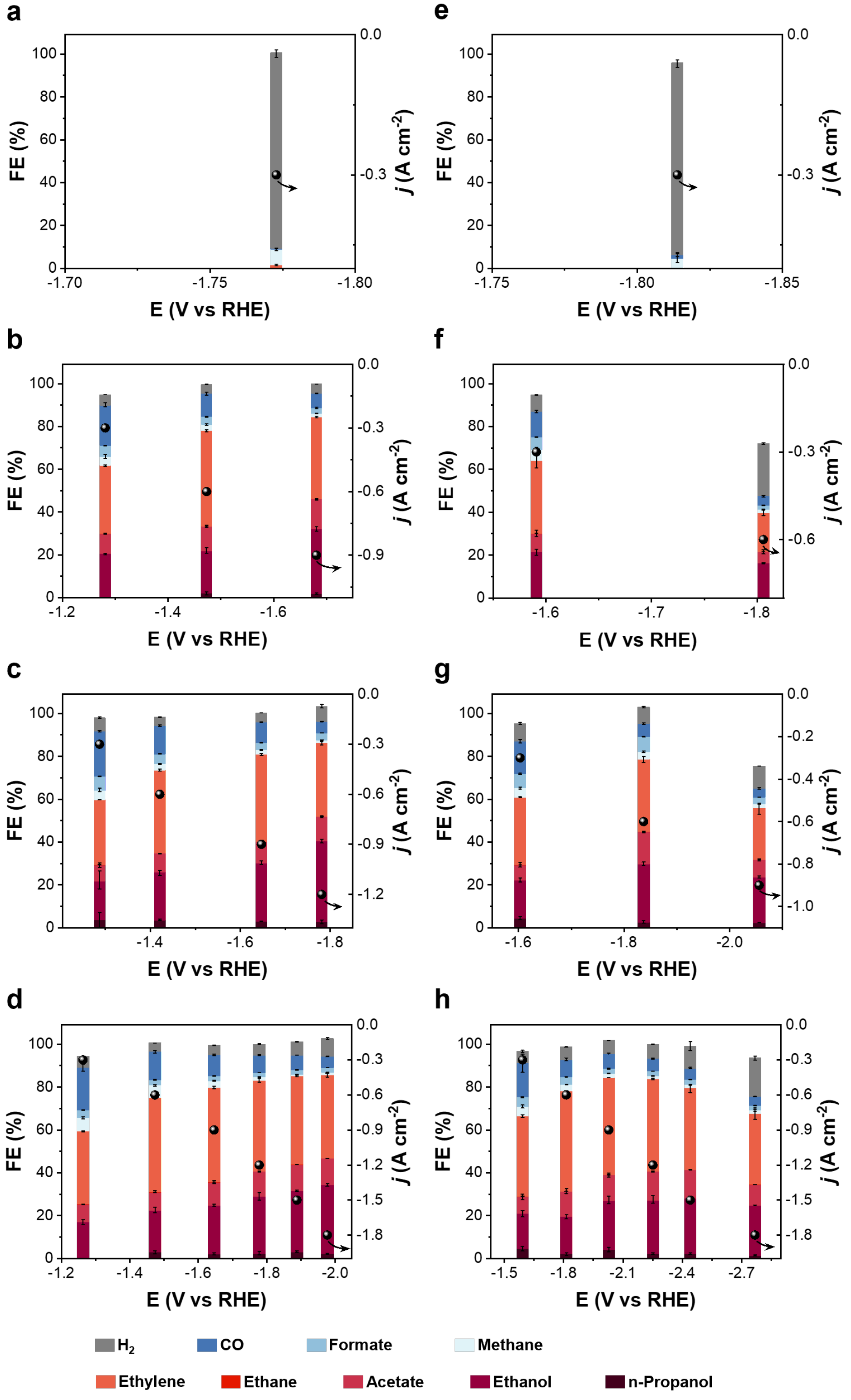

Supplementary Figure 23. CO_2RR performance of Cu-GDL in pH 6 and pH 1 electrolyte with different synthesis time. a-d, pH 6, **e-h**, pH 1, **a**,**e**, 0 min, **b**,**f**, 10 min, **c**,**g**, 20 min and **d**,**h**, 30 min. Error bars indicate s.d. (n = 3 replicates).

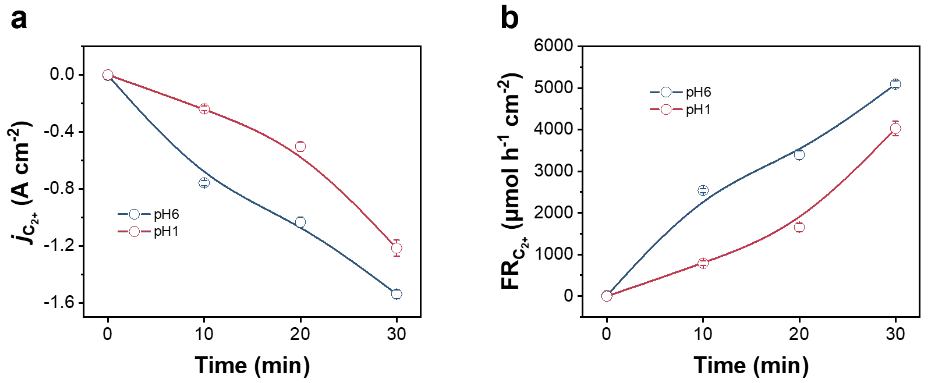

Supplementary Figure 24. CO_2RR performance of Cu-GDL for the formation of C_{2+} compounds as a function of different synthesis time. a, C_{2+} partial current density ($j_{C_{2+}}$) and **b**, C_{2+} formation rate ($FR_{C_{2+}}$). Error bars indicate s.d. (n = 3 replicates).

The calculation of $j_{C_{2+}}$ and $FR_{C_{2+}}$ can be referred to Equation 12 and Equation 13.

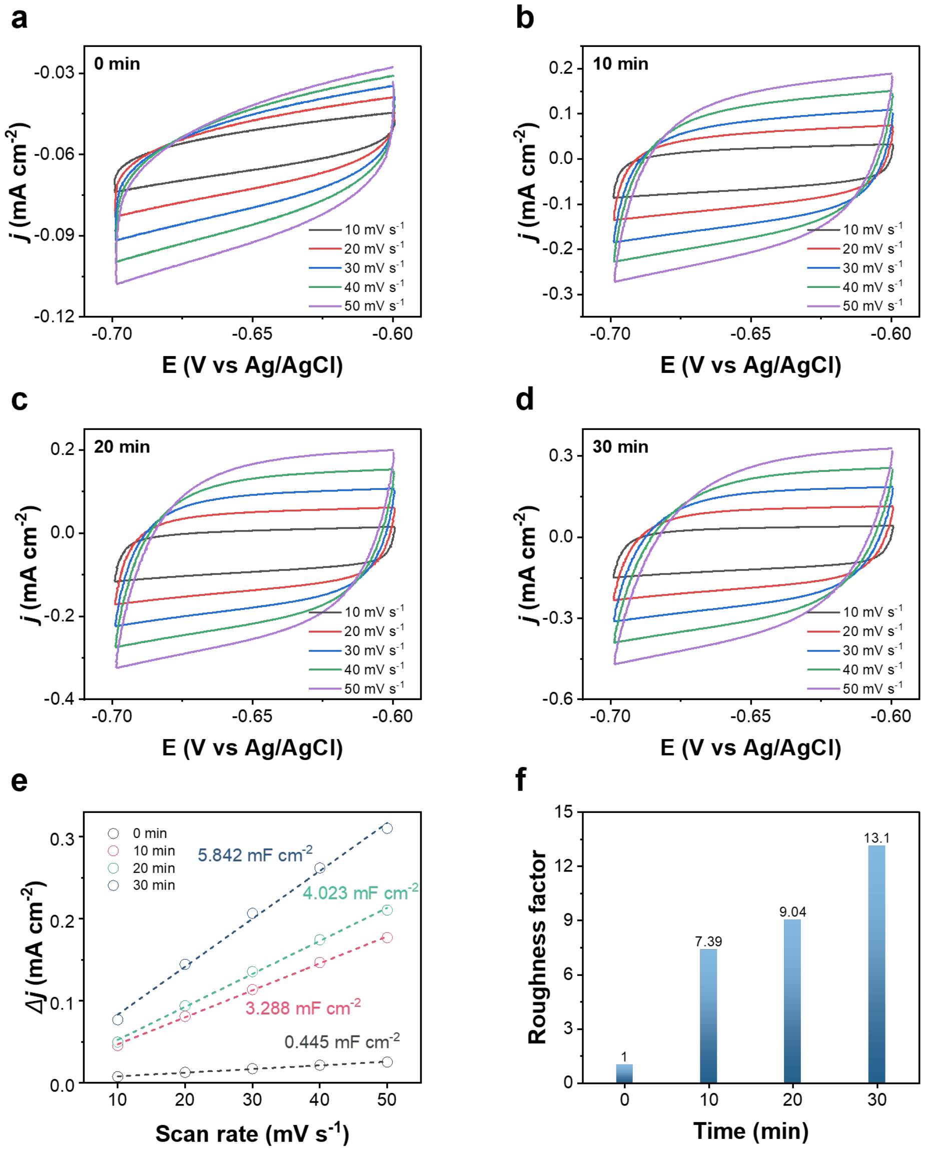

Supplementary Figure 25. Electrochemical surface areas (ECSA) and roughness factors (rf) for porous Cu with different synthesis time. CV of **a**, 0 min, **b**, 10 min, **c**, 20 min, **d**, 30 min, **e**, ECSA and **f**, rf.

The ratio of the slope/0.445 gave the rf of the surface and the rf value for a Cu foil surface is defined as 1.

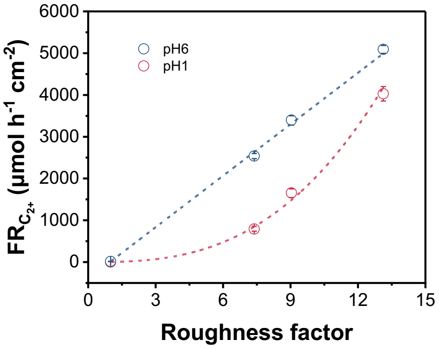

Supplementary Figure 26. C_{2+} formation rate as a function of roughness factor in pH 6 and pH 1 electrolyte. Error bars indicate s.d. (n = 3 replicates).

Based on the relationship between $FR_{C_{2+}}$ and rf (Supplementary Figure 26 and Figure 4b), it could be observed that there was a linear relationship between $FR_{C_{2+}}$ and rf at pH 6, while $FR_{C_{2+}}$ showed linear response to rf^2. The corresponding slopes were calculated from these linear responses.

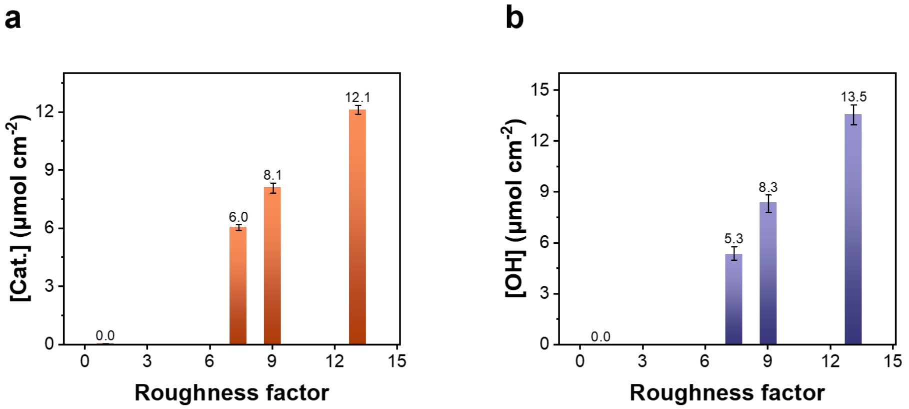

Supplementary Figure 27. Composition analysis of the electrode. a, Concentration of catalytic sites ([Cat.]) and **b**, OH ([OH]) for each roughness factor. Error bars indicate s.d. (n = 3 replicates).

The concentration of catalytic sites [Cat.] corresponding to the rf of the electrode was calculated by substituting the slope obtained at pH 6 (Supplementary Figure 26) into Equation 8, as shown in Figure 27a. Similarly, the square of the catalyst concentration $[Cat.]^2$ corresponding to the rf of the electrode was obtained by using the slope value obtained at pH 1 (Figure 4b) in Equation 8. Considering that the OH concentration [OH] is related to [Cat.] since the OH originates from the catalyst, [OH] was calculated by dividing $[Cat.]^2$ by [Cat.] obtained from Supplementary Figure 27a, as shown in Supplementary Figure 27b.

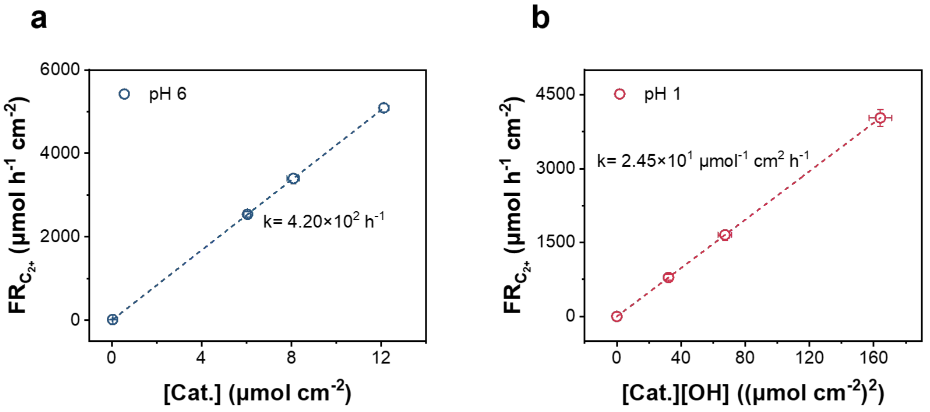

Supplementary Figure 28. C_{2+} formation rate analysis. C_{2+} formation rate ($FR_{C_{2+}}$) as a function of **a**, concentration of catalytic sites ([Cat.]) and **b**, the multiplication of [Cat.] and OH concentration ([Cat.][OH]). Error bars indicate s.d. (n = 3 replicates).

Based on the data obtained from Supplementary Figure 24 for $FR_{C_{2+}}$ and from Supplementary Figure 27 for [Cat.] and [OH], these values are inserted into Equations 8 and 9 to calculate the reaction rate constants corresponding to the respective pH. As a result, the reaction rate constant towards **Cu-GDL** is 4.20×10^{2} h^{-1} at pH 6 (first-order reaction), and 2.45×10^{1} μmol^{-1} cm^{2} h^{-1} at pH 1 (second-order reaction).

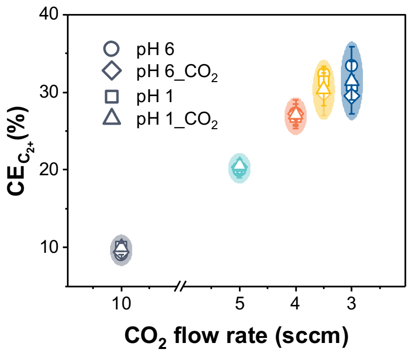

Supplementary Figure 29. $CE_{C_{2+}}$ as a function of CO_2 flow rate in different electrolyte at −0.5 A cm^{-2}. Error bars indicate s.d. (n = 3 replicates).

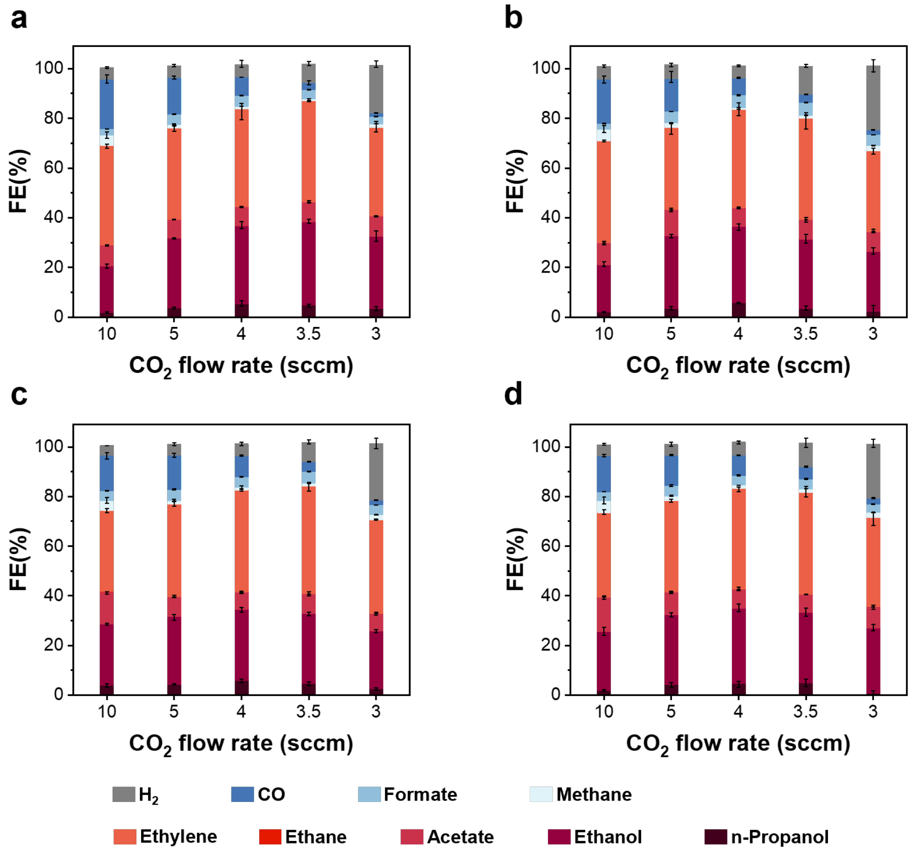

Supplementary Figure 30. CO_2RR performance of Cu-GDL with different electrolyte and CO_2 flow rate at −0.5 A cm^{-2}. a, 1 M KCl + HCl, pH 6, **b**, CO_2 saturated (**a**), pH 3.8, **c**, 1 M KCl + HCl, pH 1 and **d**, CO_2 saturated (**c**), pH 0.9. Error bars indicate s.d. (n = 3 replicates).

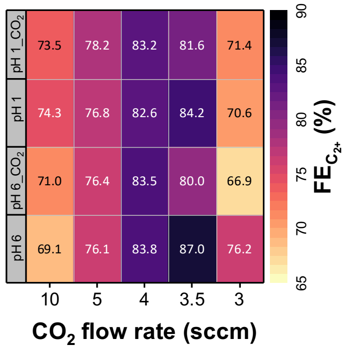

Supplementary Figure 31. $FE_{C_{2+}}$ of Cu-GDL with different electrolyte and CO_2 flow rate at −0.5 A cm^{-2}.

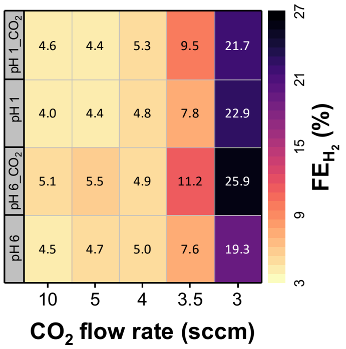

Supplementary Figure 32. FE_{H_2} **of Cu-GDL with different electrolyte and** CO_2 **flow rate at** -0.5 **A cm**$^{-2}$**.**

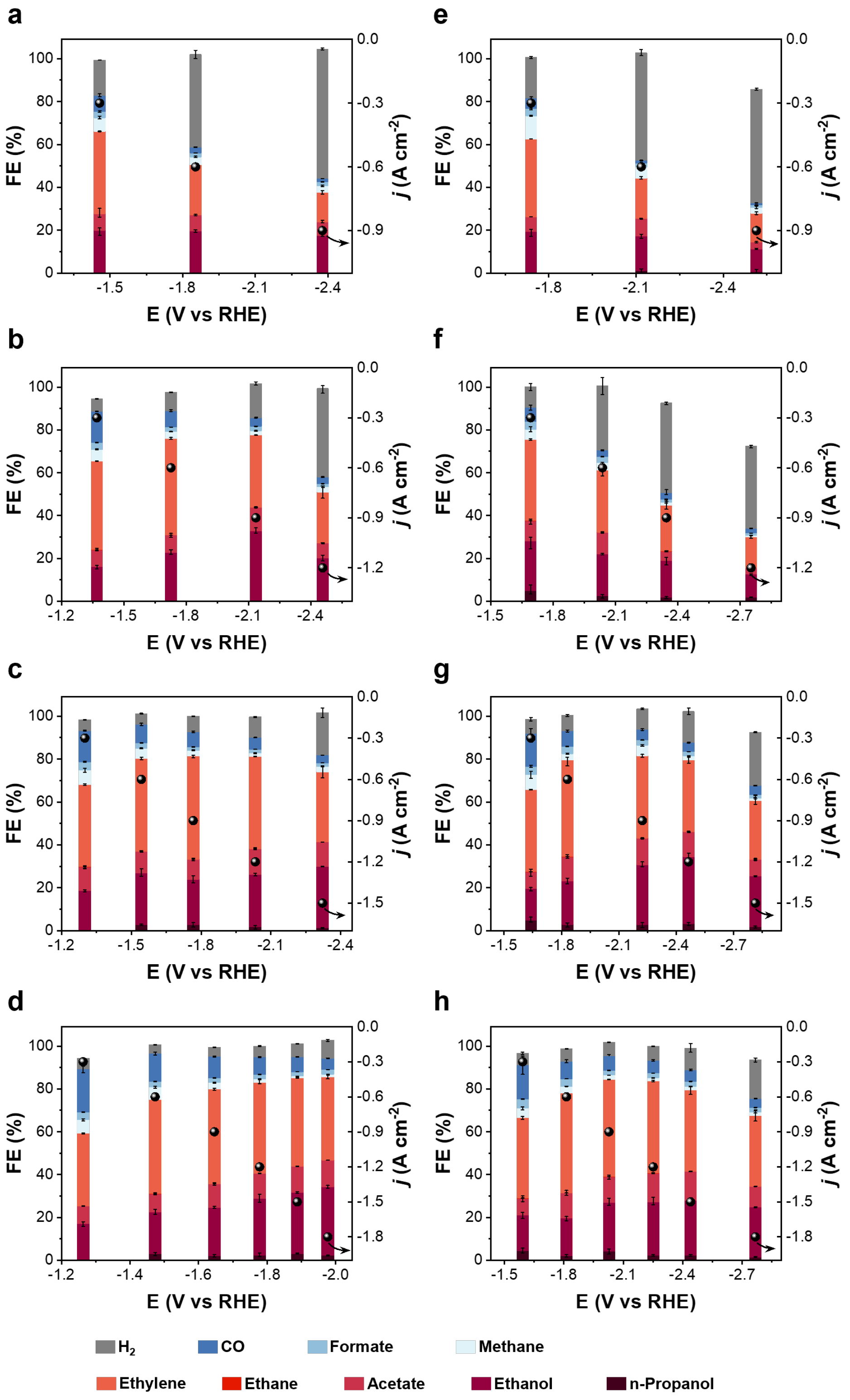

Supplementary Figure 33. CO_2RR performance of Cu-GDL with different CO_2 concentration. a-d, pH 6, **e-h**, pH 1. **a**,**e**, 25%, **b**,**f**, 50%, **c**,**g**, 75% and **d**,**h**, 100%**.** Error bars indicate s.d. (n = 3 replicates).

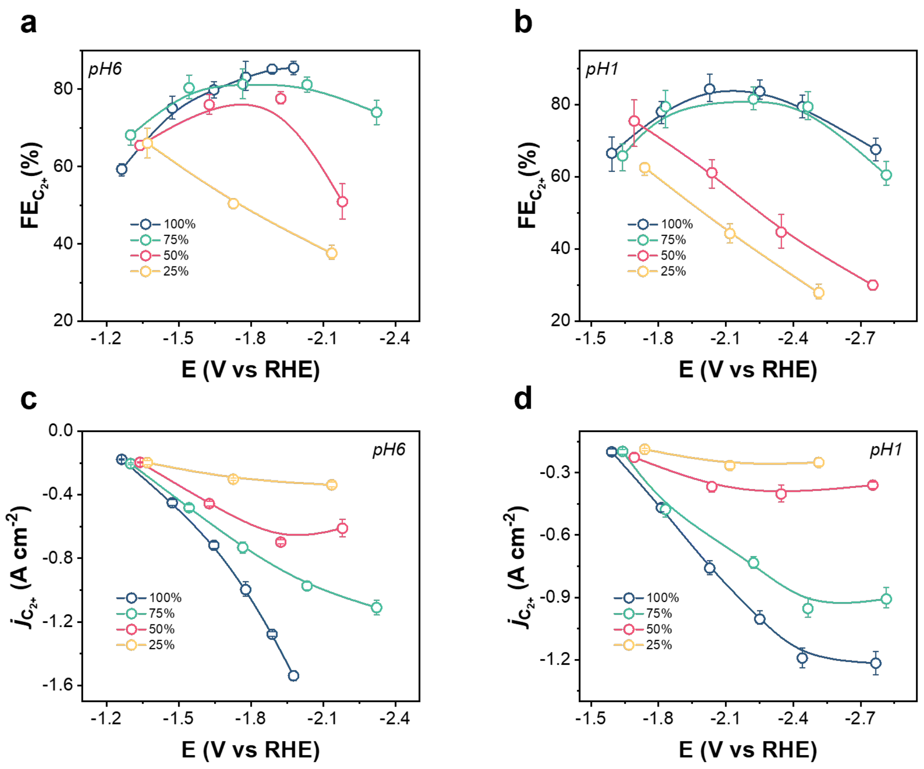

Supplementary Figure 34. CO_2RR performance of Cu-GDL with different CO_2 concentrations. a-b, pH 6 and **c-d**, pH 1. **a**,**c**, $FE_{C_{2+}}$ and **b**,**d**, $j_{C_{2+}}$. Error bars indicate s.d. (n = 3 replicates).

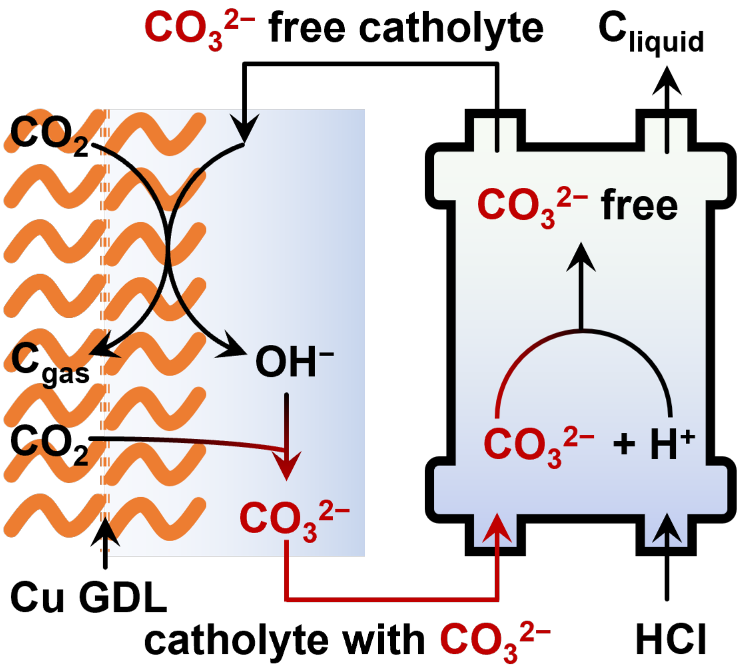

Supplementary Figure 35. Schematic of an automatic electrolyte renewal system (circulating electrolyte is 1 M KCl + 1M HCl, maintain pH≈1)

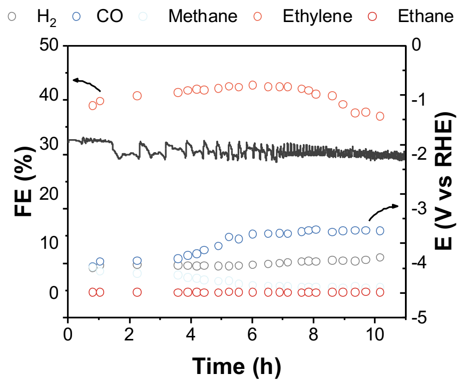

Supplementary Figure 36. The CO_2RR stability measurement in electrolyte of pH≈1 with an applied *j* of −600 mA cm^{-2}.

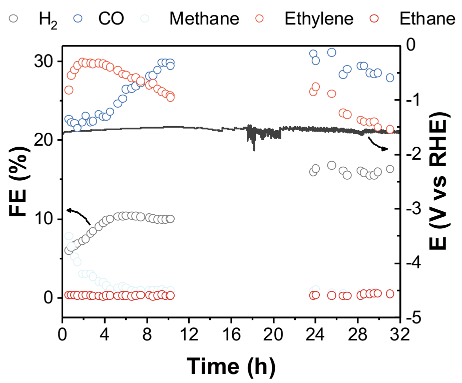

Supplementary Figure 37. The CO_2RR stability measurement in electrolyte of pH≈1 with an applied *j* of −200 mA cm^{-2}.

Approximately 18 hours into the reaction, noticeable fluctuations in potential became evident, which could potentially be attributed to a weakening of the electrode's superhydrophobic properties. Despite this observation, after running for 24 hours, we continued to observe uncompromised ethylene selectivity. In comparison to the stability test at 600 mA cm^{-2} (Supplementary Fig. 36), the ethylene selectivity exhibited a decrease and CO selectivity showed an increase during the first 10 hours at 200 mA cm^{-2}. This phenomenon can be attributed to the trend of decreasing applied potential as the reaction progresses, as indicated by the potential curve, which favors CO production at lower potentials.

Supplementary Table 1. The theoretical maximum SPCE of the corresponding product.

Product	CO_2RR	Carbonate formation	SPCE (%)
Formic acid	$CO_2 + 2H_2O + 2e^- \rightarrow HCOOH + 2OH^-$	$CO_2 + 2OH^- \rightarrow H_2O + CO_3^{2-}$	50.0
Carbon monoxide	$CO_2 + H_2O + 2e^- \rightarrow CO + 2OH^-$	$CO_2 + 2OH^- \rightarrow H_2O + CO_3^{2-}$	50.0
Methane	$CO_2 + 6H_2O + 8e^- \rightarrow CH_4 + 8OH^-$	$4CO_2 + 8OH^- \rightarrow 4H_2O + 4CO_3^{2-}$	20.0
Ethylene	$2CO_2 + 8H_2O + 12e^- \rightarrow C_2H_4 + 12OH^-$	$6CO_2 + 12OH^- \rightarrow 6H_2O + 6CO_3^{2-}$	25.0
Ethane	$2CO_2 + 10H_2O + 14e^- \rightarrow C_2H_6 + 14OH^-$	$7CO_2 + 14OH^- \rightarrow 7H_2O + 7CO_3^{2-}$	22.2
Ethanol	$2CO_2 + 9H_2O + 12e^- \rightarrow C_2H_5OH + 12OH^-$	$6CO_2 + 12OH^- \rightarrow 6H_2O + 6CO_3^{2-}$	25.0
Acetic acid	$2CO_2 + 6H_2O + 8e^- \rightarrow CH_3COOH + 8OH^-$	$4CO_2 + 8OH^- \rightarrow 4H_2O + 4CO_3^{2-}$	33.3
n-Propanol	$3CO_2 + 13H_2O + 18e^- \rightarrow$ n-$C_3H_7OH + 18OH^-$	$9CO_2 + 18OH^- \rightarrow 9H_2O + 9CO_3^{2-}$	25.0

Supplementary Table 2. Theoretical maximum SPCE of CO_2 at a flow rate of 3.5 sccm.

	Theoretical maximum SPCE (%)			
Product	pH 6	pH 6_CO_2	pH 1	pH 1_CO_2
Formic acid	5.13	6.96	6.14	5.32
Carbon monoxide	3.72	4.54	5.20	6.53
Methane	0.08	0.17	0.140	0.19
Ethylene	9.18	9.25	9.45	9.08
Ethane	0.002	0.001	0.002	0.002
Ethanol	7.53	6.34	6.14	6.27
Acetic acid	3.56	3.53	3.44	3.21
n-Propanol	1.09	0.80	0.99	1.08
Total	30.29	31.59	31.49	31.68

Equation 17 can be used to calculate the SPCE for each target product in Supplementary Table 2. "Total" in Supplementary Table 2 represents the total SPCE associated with the CO_2 conversion to products. The difference between the experimentally obtained SPCE from Figure 4d and the "Total" in Supplementary Table 2 represents the fraction of CO conversion into products beyond the theoretical maximum. The remaining fraction that has not been converted into products is the fraction contributing to carbonate formation.

References

1. Ma, H.Y. et al. Electrochemical investigation of dynamic interfacial processes at 1-octadecanethiol-modified copper electrodes in halide-containing solutions. *Electrochim. Acta* **48**, 4277-4289 (2003).
2. Dilimon, V.S., Denayer, J., Delhalle, J. & Mekhalif, Z. Electrochemical and spectroscopic study of the self-assembling mechanism of normal and chelating alkanethiols on copper. *Langmuir* **28**, 6857-6865 (2012).
3. Sun, M., Staykov, A. & Yamauchi, M. Understanding the Roles of Hydroxide in CO2 Electroreduction on a Cu Electrode for Achieving Variable Selectivity. *ACS Catalysis* **12**, 14856-14863 (2022).
4. Niaura, G. Surface-enhanced Raman spectroscopic observation of two kinds of adsorbed OH− ions at copper electrode. *Electrochim. Acta* **45**, 3507-3519 (2000).
